# Socio-Psychological Functions of Men and Women Triathlon Participation

**DOI:** 10.3390/ijerph182211766

**Published:** 2021-11-09

**Authors:** Joanna Poczta, Nuno Almeida, Ewa Malchrowicz-Mośko

**Affiliations:** 1 Faculty of Sport Sciences, Poznan University of Physical Education, 61-871 Poznań, Poland; malchrowicz@awf.poznan.pl; 2 CiTUR, School of Tourism and Maritime Technology, Polytechnic of Leiria, 2411-901 Leiria, Portugal; nunoalmeida@ipleiria.pt

**Keywords:** motives for sports participation, triathlon, women’s sport, men’s sport, non-elite sport

## Abstract

Motivations to run marathons have been recognised by many researchers, but few have paid attention to triathletes. Mass triathlon participation is a new trend, which manifests itself as a human need to invoke strong emotions and seek them in difficult sports, as well as to travel to participate in such events. Therefore, the main goal of this study was to recognise the motivations to participate in triathlons among men and women respondents, and to evaluate the differences between them. The empirical research among triathletes (*n* = 1141) recognised the motives for participation in mass triathlon sporting events in accordance with four types of orientation: social, experience, factual, and result. Most important conclusions resulting from the conducted research indicate that women significantly more often displayed the will to feel unity and integration, as well as the desire to gain recognition in the eyes of others, as compared to men. For men, the desire to feel equal was significantly more important than for women. Both men and women indicated the desire to maintain good physical condition and health, which turned out to be a significant factor. For men, Group B—specifying the experience orientation, was deemed the most important, while for women the most important group of motives was Group D—specifying the result orientation.

## 1. Introduction

The development of mass sporting events gives rise to questions regarding the motivations to participate in them. The literature on the subject mainly focuses on research results concerning mass street running in the context of its importance for sports events and the development of sports tourism, running motivation and health implications for the runners [1,2,3,4,5,6]. This may be important in the context of research on the Theory of Participation in Recreational Sports, as participation behaviour in sports can be viewed as a choice or expression of firstly, individual motives; secondly, individual attitudes and preferences, and thirdly, skills that lead to participation in a specific context related to the organisation of the event [7]. Borgers and co-authors suggested that trends in LTSP (leisure-time sports participation) should be perceived as the interplay of social processes, traditions and individual activities, as well as organisational cultures [7]. Different groups of participants are expected to have different LTSP preferences and themes, which may be reflected in different participation patterns in sports. Furthermore, to understand the changes in participation in sports, one should give consideration to extending LTSP capabilities to a variety of programs designed for different target groups, as well as policies and strategies promoting sports as an instrument of health benefit or social inclusion [8,9]. LTSP takes place in a socio-cultural context and is structured within an organisation, as well as is associated with prevailing social and cultural values, perceptions, rules, routines, symbols and procedures [10,11]. Therefore, it is suggested that institutions change over time due to the developing values and norms in society or community [12]. When institutional logic changes, organisations must adapt and recognise these new values for them to be accepted by the relevant actors or members of the organisation [13,14]. Many authors, like Nikolaidis and co-authors [5], Ogles and Masters [15], Pelletier and co-authors [16], Vallerand [17] or Briere [18] have studied the motives for participation in sports, and many research tools have been created and utilised not only among athletes in good health condition. The Sport Motivation Scale (SMS) is a tool developed by Pelletier and co-authors [16]. The purpose of SMS is to determine the level of “internal motivation, external motivation and lack of motivation” of a person functioning in the sports environment, and to identify the source of such person’s motivation [17,19,20,21]. This method is highly acclaimed in the world of sports psychology literature [16]. The SMS scale is used in sports psychology and mass research on the determinants of recreational activity in France and Greece [18,19,20,21,22,23]. It was adopted for research in Greece and Italy [23,24,25].

Numerous authors focused on research that directly related to the triathlon. For example, Raggiotto et al., as a result of their research, distinguished three groups (clusters) of triathletes: (1) indifferent—treating the competition as a hobby, (2) amateurs—for whom the results are important, but the triathlon does not play a key role in their lives, (3) professionals—who show high involvement in competing in the triathlon [26]. Mullin et al. developed a division of triathletes in sports competitions by their involvement [27]. Furst et al. investigated the motivation of disabled athletes to participate in triathlons [28]. Lovett, Barnes and Marley investigated the motives to participate in the triathlon [29]. Croft et al. constructed the levels of motivation in a group of elite and non-elite Australian triathletes [30]. This instrument was used as an altered version of The Motivation of Marathoners Scale [31]. They found that the main motivation for competing in the event was the achievement of personal goals, as well as orientation towards competition and health, which are the highest motivations for triathletes. Whereas according to the research results of Croft et al., the variables, the meaning of life from the psychological motives category and psychological coping, had the least influence on motivation [30]. Saayman et al. investigated motivation to identify different market segments of triathletes participating in Ironman South Africa [32]. Factor analysis showed that triathletes were motivated by seven factors, including challenges, internal struggle, health and fitness, internal achievement and control, news of the event, belonging to a group and socialisation, and finally respect and risk. Based on this division of motives, three different groups of triathlon participants were identified: devotees, enthusiasts, and aspirants. Although these participants have different motives, the only factor that exists is significant age differences, with no other socio-demographic characteristics. Wicker et al. examined the characteristics of triathlon participants and divided the entire market between Germany into smaller consumer segments. Data on triathletes’ behaviour, psychograph (lifestyle) and demographics were collected through an online survey. Lifestyle segmentation was performed using the k-means cluster analysis, which suggested three clusters. These are serious chasers, sports lovers and socialites, depending on their activities and interests in their free time. The Chi2 test revealed significant differences between the clusters in terms of age, gender, years of participation, seniority and expenses [33]. The test results of Wicker are not similar to the results of Saayman, who found the only statistically important difference in age. Lopez-Fernandez and co-authors examined the relationship between sex and motivation in triathletes, utilising a multidimensional measurement of motivation in sports. This research involved the use of the Sport Motivation Scale, which consists of a multi-dimensional measurement of the assessment of different types of athletes’ motivation to practice sports. The results showed a significant difference in motivation depending on the sex, as women received lower scores; however, the motivation scores overall were very low. The results were not dependent on occupation or age. Men and women participating in the triathlon at the international level have similar motivational profiles [34]. According to Roethenbaugh’s research, on average, 30% of participants in triathlon competitions are women. The most numerous age category for both men and women is the 34–40 range [35,36]. Therefore, the secondary goal of these studies was to identify the motivation to participate in the triathlon between men and women. From the perspective of the conducted research, demonstrating differences between sex in motivations to start in the triathlon turned out to be not only interesting but also niche. Such research has not yet been carried out in Poland.

## 2. Goals of the Study

The main objective of the study was to investigate the motives for participating in mass triathlon events. The secondary goal was to identify the motivation to participate in the triathlon between men and women and to assess the differences between them. The diagnosis of the motives for participating in a sports event was tested according to four types of orientation: social, experience, factual and results; to check what benefits for well-being and self-improvement are brought by participation in a triathlon as a mass sports event.

## 3. Materials and Methods

### 3.1. Design of the Research

The division of Freyer and Gross [37], who distinguished four types of orientation among the motives of participation in sporting events, was the basis for the development of the author’s questionnaire survey of motives for participation in running events. The authors distinguished four main types of orientation among motives for participation in sports events: (a) social orientation, which is focused on the relationships of visitors to each other; (b) sensation-seeking orientation, which is most often concerning the positive experiences, in the form of, for example, relaxation, which is a kind of compensation for the hardships of everyday life; (c) factual orientation/sports discipline orientation, referring to the sports events themselves and their specificity; in this case, to the specificity of running, and (d) result orientation, which is triggered by the need to identify with success, and in the case of failure, by the need to show sympathy and solidarity. Since our authorship research tool was validated during the 5th and 6th Poznan Half Marathon, the questionnaire and appropriate details concerning data selection were provided by a single researcher.

The study was conducted to evaluate the reasons for attempting a triathlon, the perceived outcomes from running, swimming and cycling, and participants’ experiences, including the development of feelings of deep personal awareness and satisfaction. The authors decided to conduct the research using a diagnostic survey method with the use of a standardised interview technique between people who declare to have participated in a triathlon one or more times.

Data collection took place from January until March 2021. The online survey was anonymous, voluntary and confidential. The sample consisted of 1141 triathletes: 721 males and 420 females. The limitation of the research was the COVID 19 pandemic, which caused the cessation of mass sports events. We were unable to conduct the interviews in person and meet the respondents. All respondents first declared participation in the triathlon, and we reached them with the help of the organiser of the Super League Triathlon in Poznań. They were also members of triathlon societies and sports clubs. Online survey forms were sent to the organiser of the Super League Triathlon in Poland and with their cooperation, data collection was being conducted in 2021.

We tried to select the sample in a way that would ensure the best possible representativeness of the obtained results. The scheme of simple random sampling without replacement was used. In determining the number of athletes, data from the organiser of the Super League Triathlon in Poland on the expected number of participants each year of the events organised in Poland was used. In calculations, the formula for the sample size for a finite population was used. The assumption was made that the maximum error of estimate (e) at 95% confidence level should not exceed 4%. Moreover, the questionnaire was in Polish, therefore only Polish triathletes could complete it. The questionnaire had 16 questions and two parts focused on socio-demographic variables and motives for participating in sporting events. The results, consisting of different types of motives, could exceed 100% because, in each group of motives, participants could tick more than one answer (maximum of three answers).

Respondents were informed about the nature and goal of the survey. The study was conducted in conformity with the Declaration of Helsinki, with all participants treated according to the American Psychological Association’s ethics code. The Bioethics Committee at the Poznan University of Medical Sciences, Poland, confirmed that, according to national regulations, the study involves no experiments and thus does not need to undergo an ethics review.

### 3.2. Participants

A sample of 1141 triathletes: 721 men and 420 women respondents. The respondents (*n* = 1141) were mainly between 41–50 years of age (41.36%—472), 36–40 years of age (40.6%—343), and 25–36 years of age (*n* = 160, 14.02%). Respondents with higher education constituted the vast majority 80.63% (920), and 88.95% (1015) were professionally active. The socio-demographic characteristics of the respondents are presented below (Table 1).

Among the surveyed men (*n* = 721), the majority of runners were aged between 41 and 50 (51.17%—369), and then between 36 and 40 (20.94%—151). Men with higher education constituted the vast majority—78.77% (568), while 10.67% (77) possessed incomplete higher education. A great percentage—94.03% (678)—were professionally active. Most of the women (191) were aged 36–40 (45.47%). Among the surveyed women, 352 completed higher education (83.80%). Over 80.00% (336) of women participants were professionally active. The research shows that younger women take part in triathlons more often than men.

### 3.3. Data Analysis

Descriptive statistics (percentages, means, and standard deviations) were calculated for all of the variables. To verify whether a respondent’s sex had a significant impact on their motives for participating in the event, chi-square tests were carried out, and, with significant results, the phi coefficients were calculated to determine the strength of this relationship. Describing the strength of association, it is assumed that: >0.5—high association, 0.3 to 0.5—moderate association, 0.1 to 0.3—low association and 0 to 0.1—little if any association. Statistical significance was set at *p* ≤ 0.05. All of the statistical analyses were conducted using Statistica Software 10.0 (StatSoft Inc., Cracow, Poland, 2011).

## 4. Results

The analysis indicates that in the scope of social orientation motives (Table 2), the main factor for taking part in the triathlon was the desire to feel unity and integrate with other people (320 triathletes). The desire to gain recognition in the eyes of others was important for 151 respondents, while the next place was taken by the sense of belonging to the subculture of runners (109). Athletes (90) marked that they wanted to feel equality with other people during the race, but interestingly, not a single woman chose this motive (Table 2).

To verify whether a respondent’s sex had a significant impact on their motives for participation in the event, chi-square tests were carried out, and in the case of significant results, the phi coefficients were calculated to determine the strength of this relationship. The results of the analysis showed a significant relationship between sex and the desire for a sense of unity and integration, the desire for a sense of equality gaining recognition and belonging to a subculture as a motive for participating in an event. Women (32.1%) significantly more often indicated the will to feel unity and integration as compared to men (25.7%), but the strength of this correlation is very small (phi = 0.07). Women (20.7%) also more frequently indicated the desire to gain recognition in the eyes of others as compared to men (8.9%), with a small effect (phi = 0.17). In the case of men (12.5%), the desire to feel equal was significantly more important than for women (0%), with a small effect (phi = 0.22).

Table 3 presents motives within the scope of experience orientation. The desire to experience strong emotions associated with participation in the triathlon was the most important motive in Group B. This answer was declared by 65.73% (750) of the respondents. The desire to have fun was important for 51.79% of surveyed athletes (591). The desire to experience a unique mood during the event was declared by 51.53% (588) of the respondents. Last but not least, there was also the desire to get away from everyday life (important to 268 athletes (23.48%)) and have some enjoyable leisure time, which was important to 213 people (18.66%).

To verify whether a respondent’s sex had a significant impact on their motives for participating in the event, chi-square tests were carried out and, and in the case of significant results, the phi coefficients were calculated to determine the strength of this relationship. All dependencies, apart from the desire to express joy and the attractiveness of the city, turned out to be significant, with very little or no effect.

The analysis of all surveyed athletes shows that (Table 4), in the scope of factual orientation, more than three-quarters of the competitors—78%—wanted to develop their passion, but this was more important for men (84.2%). Over half of competitors (54.42%) took part in the event due to the attractiveness of the sports part of the triathlon (including an attractive route). However, these motives were more often chosen by men (61.7%) than women (41.9%).

To verify whether a respondent’s sex had a significant impact on their motives for participating in the event, chi-square tests were performed and, and in the case of significant results, the phi coefficients were calculated to determine the strength of this relationship. All relationships turned out to be significant, with little effect.

The most frequently indicated motives from group D (in terms of the result orientation), and at the level of 87.37% (997) of all indications, was the desire to test themselves (Table 5). The motive associated with the desire to achieve the avowed goal was also highly rated, as it was indicated by 77.65% (886) of the surveyed athletes. Participation in a sports competition was more important for women (52.4%) than for men (44%). The desire to achieve a high (international) rank in this sports event was more important for women as well, and this motive was statistically significant (*p* ≤ 0.05). Chi-square tests were carried out and, and in the case of significant results, the phi coefficients were calculated to determine the strength of this relationship.

In the case of failure to meet the assumption regarding the expected numbers (too small a sample), the Fisher exact test was used (none of the motives, Table 5). Correlations between sex and the willingness to participate in sports competition, the desire to win and failure to indicate any of the motives in this group turned out to be significant, with a very weak or weak effect.

The desire to maintain good physical condition and health (Table 6) was reported by 93.2% (672) of the surveyed men, and by 88.8% (373) of the women.

As in the previous cases, to verify whether a respondent’s sex had a significant impact on their motives for participation in the event, chi-square tests were carried out and the phi coefficients were calculated to determine the strength of this relationship. Both relationships turned out to be significant, with very little or no effect.

The respondents were also asked to indicate which group of motives is the most important for them (Table 7).

Group D—specifying result orientation, was indicated by a major number of men and women (37.68%), but in the case of men, a more important group was Group B—specifying experience orientation (37.58%). In turn, Group D was more important for women (38.33%).

## 5. Discussion

Our findings indicate that in Group A (the scope of social orientation motives) the main factor for taking part in the triathlon was the desire to feel unity and integration with other people. Women significantly more often indicated the will to feel unity and integration, as well as the desire to gain recognition in the eyes of others as compared to men. For men, the desire to feel equal was significantly more important than for women. Whereas, in Croft et al. research results, the variables such as life meaning, seen from the psychological motives category, and psychological coping had the least influence on motivation [30].

The desire to experience strong emotions associated with participation in the triathlon and have good fun was the most important motive in Group B (the scope of experience orientation), while the desire to experience the unique mood during the whole event and to have fun was more important for women. All dependencies, apart from the desire to express joy and the attractiveness of the city, turned out to be significant.

Group C (the scope of factual orientation) shows that respondents wanted to develop their passion and they took part in the event due to the attractiveness of the sports part of the triathlon (including an attractive route). These motives had more indications by men than by women. All relationships turned out to be significant, with little effect.

Among the motives from Group D (in terms of the result orientation), the most frequently indicated was the desire to test themselves and the desire to achieve the avowed goal. Participation in sports competition and the desire to win a high (international) rank in this sports event was more important for women. This motive was statistically significant (*p* ≤ 0.05). These results correspond with the research reports published by Croft et al. where the main motivation for completing the event was personal goal achievement, competition and health orientations [30]. In our research, both men and women respondents, when asked about other motives for participation in a half marathon, indicated the desire to maintain good physical condition and health, which turned out to be significant. For men, the most important group of motives was Group B—specifying the experience orientation, but for women, the most important group of motives was Group D—specifying the result orientation. However, in total, Group D (motives within the scope of result orientation) received the largest number of indications. Lopez-Fernandez and co-authors, who examined the relationship between sex and motivation in triathletes utilising a multidimensional measurement of motivation in sports, showed a significant difference in motivation depending on sex. In their research, women received lower scores; and although the motivation scores were very low, both men and women participating in the triathlon at the international level had similar motivational profiles [34].

These findings relate to our previous research on motivation to participate in mass running events. We have found that the most significant differences between male and female motivations were the desire to get away from everyday life and the prevailing fashion, as well as these motives, were more important for women than for men. The desire to win was not important for either sex. The need to experience strong emotions during the race, the need to feel integrated and unified with other runners and the desire to test themselves were not only important for men but also women [1]. Comparative analysis of the most important results from previous half-marathon research and those present showed that the most often indicated motives for participation in the events both by surveyed men and women turned out to be the desire to maintain good physical condition and health. The results between triathlon participants showed that the desire to test themselves, develop passion and achieve an avowed goal is more important for men than for women. The results were similar for half marathon runners. The desire to feel strong emotions associated with participation in a sporting event, and have fun are more important for women than for men in current triathlon research. Half marathon results were different. These motives were more often important for men. Similarly, the desire to experience the unique mood during the whole event was more important for women in both groups of respondents—triathlon and half marathon participants. What is interesting, all motives in Group B—motives within the scope of experience orientation, were more important for women participating in the half marathon, but more important for men participating in the triathlon. For both men and women, half marathon and triathlon athletes, the most important motivation was the desire to test themselves and achieve the avowed goal (result orientation). Research in terms of sex and motivational differences has been conducted by many authors, although it concerned mass running events. First, Ogles and Masters found out that the most common motivations for running among women are social needs and good physical condition, whereas but men more often want to compete and achieve success [15]. Summers and co-authors discovered that women are looking for the opportunity to meet new people [38]. These results are similar to studies among triathletes.

Research of Saayman et al. on triathletes participating in Ironman in South Africa shows that triathletes were motivated by seven motivators (challenge; internal struggle; health and fitness; internal achievement and control; news of the event; belonging to a group and socialisation; respect and risk), and based on this division of motives, three different groups of triathlon participants were identified: devotees; enthusiasts, and aspirations [32]. Wicker et al. examined the characteristics of triathlon participants and named them: Serious chasers, sports lovers and socialites, depending on their activities and interests in their free time [33].

Running in marathons and triathlons is a behaviour that has seen unprecedented growth, also in Poland [6,15,39]. Authors like Sharkey or MacNeill claim that during the past two decades, running efficacy in reducing the risk of problems like diabetes, obesity, cardiovascular diseases or depression has been repeatedly demonstrated, a fact that is strictly connected with the popularity of running [40,41]. In addition to health values, running provides to its participant’s socio-cultural values, the needs of belonging to a group and enjoying fulfilling free time. People practice running and take part in half marathons to demonstrate a high demand for stimulation (sensation seeking) [4]. The need to achieve a high quality of life and the feeling of joy provided by an active lifestyle are conditioned not only by motivational processes. They are also related to the level of satisfaction of mental needs, and these in turn determine the achievement of the level of satisfaction with physical fitness. That is why nowadays people take up the challenges related to the sports lifestyle and want to become amateur athletes [3,42,43].

The relationship between cultural change processes and the practice of running and the triathlon in Poland is meaningful. With the increase in leisure time and the proliferation of post-materialist values, the fundamental configuration of socio-cultural predisposition to run can be considered as a fundamental trend: the emergence of mass customisation processes. Running and triathlons are characterised by more individualised patterns and a focus on building a personal lifestyle. Among the physically active Poles, the mode of consumption and the forms of physical activity chosen are changing, and in this respect, they are heading towards western patterns. For the contemporary consumer of culture, sport and tourism, understood as elements of lifestyle, are becoming an important set of principles, rules and values.

The results show distinct segments of different athletes that can help organisers and travel destinations create products and services that complement the participants’ motives. This could eventually lead to a more competitive and sustainable event.

## 6. Conclusions

The results of our current research prove that participation in such a mass sports event like the triathlon brings together people with high quality of life, conscious training plan and expenses related to their equipment. According to the theories of cultural change and individualisation claim, the practice of mass sporting events participation is more widespread among younger individuals and those with a higher educational level. People participate in sporting events not only for physical activity but also for socio-psychological effects. Experienced runners and triathletes need comprehensive physical activity and experience strong emotions, which are at the top of the hierarchy of post-modern human needs. The research was based on the consideration of running and triathlon participation as a physical activity that emerges and develops in the context of the growing diffusion of post-materialist values, which have characterised western societies in the past few years.

Among the physically active Poles, the mode of consumption and the forms of physical activity chosen are changing, and in this respect, they are heading towards western patterns. For the contemporary consumer of culture, sport and tourism understood as lifestyle elements, become an important set of principles, rules and values. Polish society has undergone a dynamic social change, the important aspect of which is the political, economic and socio-cultural transformation that began in 1989, which is strictly connected with post-accession migration from Poland. The post-materialist orientation fosters a distancing from federated sport and competition, a preference for sports practices directed toward the maintenance or achievement of good physical condition, well-being and appearance, as well as outdoor exercise in natural settings. Through abiding and cultivating these principles, premises for multidimensional human development, both in physical and spiritual spheres, can be created.

Our research shows that all actions are guided by a specific motivation, which gives meaning to what we do. This motivation differs between men and women. These are the most interesting aspects of the results of our research. This is confirmed by the participants. The basis for finding the motivation to take part in the triathlon is setting a goal and appropriate motivation. The triathlon is no exception. This is confirmed by the participants. The basis for finding motivation is setting a goal. Participation in a triathlon is a huge challenge that many competitors dream about. Participants in this strenuous discipline combining swimming, running and cycling, emphasise that powerlifting is attractive for them for several reasons. The most frequently mentioned advantage of the triathlon is its diversity, which allows you to engage all muscle groups during training and the very start. Preparing to start in a triathlon requires consistency, persistence and diligence, but it is not an impossible goal.

## Figures and Tables

**Table 1:** Socio-demographic characteristics of respondents.

Socio-Demographic Characteristics	Male (*n* = 721)	%	Female (*n* = 420)	%	All (*n* = 1141)	%
Age						
18<	13	1.80	25	5.95	38	3.33
19–24	13	1.80	16	3.80	29	2.54
25–35	101	14.00	58	13.80	160	14.02
36–40	151	20.94	191	45.47	343	30.06
41–50	369	51.17	103	24.52	472	41.36
51–70	73	10.12	26	6.19	99	8.67
Education Level						
Primary education	13	1.80	12	2.85	25	2.19
Secondary education	63	8.73	27	6.42	90	7.88
Incomplete higher education	77	10.67	29	6.90	106	9.29
Completed higher education	568	78.77	352	83.80	920	80.63
Employment Status						
School pupil (<18 years)	26	3.39	12	2.85	38	3.33
University student	0	0.00	29	6.90	29	2.54
Professionally active	678	94.03	336	80.00	1015	88.95
Unemployed	0	0.00	30	7.14	30	2.62
Pensioner	16	2.21	13	3.09	29	2.54
Place of Residence						
City with over 500,000 inhabitants	16	13.56	20	16.95	41	34.5
City with 100,000–500,000 inhabitants	1	0.85	8	6.78	11	8.08
City with 10,000–100,000 inhabitants	8	6.78	19	16.1	31	22.79
City with up to 10,000 inhabitants	6	5.08	4	3.39	11	8.08
Village	14	11.86	22	18.64	42	30.88

**Table 2:** Motives within the scope of social orientation.

Group A	Men (*n* = 721)		Women (*n* = 420)		All	*p*	Phi-Coefficient
	*n*	%	*n*	%			
Desire to feel unity and integration with other people	185	25.7	135	32.1	320	0.019	0.07
Desire to feel equality during the race	90	12.5	0	0.0	90	<0.001	0.22
Prevailing fashion—participation in sports events is currently fashionable	18	2.5	14	3.3	32	0.409	-
Desire to gain recognition in the eyes of others	64	8.9	87	20.7	151	<0.001	0.17
Belonging to the subculture of runners	48	6.7	61	14.5	109	<0.001	0.13
None of the motives listed in this group	449	62.3	271	64.5	720	0.448	-

**Table 3:** Motives within the scope of experience orientation.

Group B	Men (*n* = 721)		Women (*n* = 420)		All	*p*	Phi-Coefficient
	*n*	%	*n*	%			
Desire to experience strong emotions associated with participation	453	62.8	297	70.7	750	0.007	0.08
Desire to feel the unique mood during the whole event	338	46.9	253	60.2	591	<0.001	0.13
Desire to have fun	349	48.4	239	56.9	588	0.006	0.08
Desire to have enjoyable leisure time/entertainment	172	23.9	41	9.8	213	<0.001	0.17
Desire to express happiness, e.g., resulting from winning/success	77	10.7	43	10.2	120	0.815	-
Desire to get away from everyday life	197	27.3	71	16.9	268	<0.001	0.12
I am allured by the attractiveness of the city in which the event takes place	45	6.2	30	7.1	75	0.553	-
None of the motives listed in this group	41	5.7	57	13.6	98	<0.001	0.14

**Table 4:** Motives within the scope of factual orientation.

Group C	Men (*n* = 721)		Women (*n* = 420)		All	*p*	Phi-Coefficient
	*n*	%	*n*	%			
Desire to develop a passion	607	84.2	283	67.4	890	<0.001	0.20
I am drawn by the attractiveness of the sports part of the triathlon	445	61.7	176	41.9	621	<0.001	0.19
I am drawn by the attractiveness of the extensive program of events accompanying	13	1.8	27	6.4	40	<0.001	0.12
None of the motives listed in this group	28	3.9	67	16.0	95	<0.001	0.21

**Table 5:** Motives within the scope of result orientation.

Group D	Men (*n* = 721)		Women (*n* = 420)		All	*p*	Phi-Coefficient
	*n*	%	*n*	%			
Desire to test yourself	639	88.6	358	85.2	997	0.096	-
Desire to achieve the avowed goal	570	79.1	316	75.2	886	0.135	-
Desire to participate in sports competition	317	44.0	220	52.4	537	0.006	0.08
Desire to win	52	7.2	65	15.5	117	<0.001	0.13
High (international) rank of this sporting event	31	4.3	16	3.8	47	0.688	-
None of the motives listed in this group	0	0.0	13	3.1	13	<0.001	0.14

**Table 6:** Other motives.

Group E	Men (*n* = 721)		Women (*n* = 420)		All	*p*	Phi-Coefficient
	*n*	%	*n*	%			
The desire to maintain physical condition and health	672	93.2	373	88.8	1045	0.010	0.08
Other motives	63	8.7	80	19.0	143	<0.001	0.15

**Table 7:** Significance of particular groups of motives covered by the research.

Significance of the Motives Group	Men (*n* = 721)		Women (*n* = 420)		All	%
	*n*	%	*n*	%		
Group A Motives within the scope of social orientation.	45	6.24	19	4.52	64	5.61
Group B Motives within the scope of experience orientation.	271	37.58	137	32.62	408	35.76
Group C Motives within the scope of factual orientation.	64	8.87	89	21.19	153	13.41
Group D Motives within the scope of result orientation.	269	37.31	161	38.33	430	37.68
Group E Other motives (The desire to maintain physical condition and health)	73	10.12	13	3.09	86	7.54

## Data Availability

The data presented in this study are available on request from the corresponding author. The data are not publicly available.

## References

[1] Shipway R., Jones I. (2007). Running away from home: Understanding visitor experiences and behaviour at sport tourism events. Int. J. Tour. Res..

[2] Nowak P., Chalimoniuk-Nowak M. (2015). Running tourism in Poland. Example of tourist activity of Polish marathon runners. Br. J. Educ. Soc. Behav. Sci..

[3] Malchrowicz-Mośko E., Poczta J. (2018). Running as a Form of Therapy Socio-Psychological Functions of Mass Running Events for Men and Women. Int. J. Environ. Res. Public Health.

[4] Poczta J., Malchrowicz-Mośko E. (2018). Modern Running Events in Sustainable Development—More than Just Taking Care of Health and Physical Condition (Poznan Half Marathon Case Study). Sustainability.

[5] Nikolaidis P.T., Alvero-Cruz J.R., Villiger E., Rosemann T., Knechtle B. (2019). The Age-Related Performance Decline in Marathon Running: The Paradigm of the Berlin Marathon. Int. J. Environ. Res. Public Health.

[6] Nikolaidis P.T., Chalabaev A., Rosemann T., Knechtle B. (2019). Motivation in the Athens Classic Marathon: The Role of Sex, Age, and Performance Level in Greek Recreational Marathon Runners. Int. J. Environ. Res. Public Health.

[7] Borders J., Pilgaard M., Vanreusel B. (2016). Can we consider changes in sports participation as institutional change? A conceptual framework. Int. Rev. Sociol. Sport.

[8] Bergsgard N.A., Houlihan B., Mangset P. (2007). Sport Policy: A Comparative Analysis of Stability and Change.

[9] Van Bottenburg M., Nicholson M., Hoye R., Houlihan B. (2011). Participation in Sport: International Policy Perspectives.

[10] Klostermann C., Nagel S. (2014). Changes in German sport participation: Historical trends in individual sports. Int. Rev. Sociol. Sport.

[11] Llopis-Goig R. (2014). Sports participation and cultural trends. Running as a reflection of individualisation and post-materialism processes in Spanish society. Eur. J. Sport Soc..

[12] Gilmore S., Sillince J. (2013). Institutional theory and change: The deinstitutionalisation of sports science at club X. J. Organ. Chang. Manag..

[13] Greenwood R., Hinings C.R., Whetten D. (2014). Rethinking organisations and institutions. J. Manag. Stud..

[14] Stenling C. (2013). The introduction of drive-in sport in community sport organizations as an example of organizational non-change. J. Sport Manag..

[15] Ogles B.M., Masters K.S. (1995). Obligatory running and gender: An analysis of participative motives and training habits. Int. J. Sport Psychol..

[16] Pelletier L.G., Tuson K.M., Fortier M.S., Vallerand R.J., Briere N.M., Blais M.R. (1995). Toward a New Measure of Intrinsic Mo-tivation, Ex Motivation and Amotivation in Sports. J. Sport Exerc. Psychol..

[17] Vallerand R.J. (1997). Toward A Hierarchical Model of Intrinsic and Extrinsic Motivation. Adv. Exp. Soc. Psychol..

[18] Briere N.M., Vallerand R.J., Blais M.R., Pelletier L.G. (1995). Development et validation d’une mesure de motivation intrinsfeque et extrinseque et d’amotivation en contexte sportif: L’Echelle de Motivation vis-à-vis les Sports (EMS) [Development and Vali-dation of a Measure of Intrinsic, Extrinsic, and Amotivation in Sports: The Sport Motivation Scale (SMS)]. Int. J. Sport Psychol..

[19] Deci E.L., Ryan R.M. (2008). Facilitating optimal motivation and psychological well-being across life’s domains. Can. Psychol. Can..

[20] Brown J., Neporent L. (2015). Runner’s World: The Runner’s Brain.

[21] Miller K.A., Deci E.L., Ryan R.M. (1988). Intrinsic Motivation and Self-Determination in Human Behaviour.

[22] Alexandris K., Carroll B. (1997). An analysis of leisure constraints based on different recreational sport participation levels: Results from a study in Greece. Leis. Sci..

[23] Alexandris K., Tsorbatzoudis C., Grouios G. (2002). Perceived Constraints on Recreational Sport Participation: Investigating their Relationship with Intrinsic Motivation, Extrinsic Motivation and Amotivation. J. Leis. Res..

[24] Doganis G. (2000). Development of a Greek Version of the Sport Motivation Scale. Percept. Mot. Skills.

[25] De Pero R., Amici S., Benvenuti C., Minganti C., Capranica L., Pesce C. (2009). Motivation for Sport Participation in Older Italian Athlets: The Role of Age, Gender and Competition Level. Sport Sci. Health.

[26] Raggiotto F., Mason M., Moretti A., Ciani S. (2016). The Triathlon Sport Consumer. A Segmentation Proposal.

[27] Mullin B.J., Hardy S., Sutton W.A. (2007). Sport Marketing.

[28] Furst D.M., Ferr T., Megginson N. (1993). Motivation of Disabled Athletes to Participate in Triathlons. Psychol. Rep..

[29] Lovett M., Barnes J., Marley S. (2018). An examination of the motives to participate in sprint-distance triathlon. J. Sport Behav..

[30] Croft S.J., Gray C.C., Duncan J.F. (1999). Motives for participating in triathlon: An investigation between elite and non-elite com-petitors in an Australian setting. Health.

[31] Masters K.S., Ogles B.M., Jolton J.A. (1993). The Development of an Instrument to Measure Motivation for Marathon Running: The Motivations of Marathoners Scales (MOMS). Res. Q. Exerc. Sport.

[32] Saayman M., Kruger M., Myburgh E. (2014). A motivation-based typology of triathletes. S. Afr. J. Res. Sport Phys. Educ. Recreat..

[33] Wicker P., Hallmann K., Prinz J., Weimar D. (2012). Who takes part in triathlon events? A application of lifestyle segmentation to triathlon participants. Int. J. Sport Manag. Mark..

[34] López-Fernández I., Marbán R.M., Fernández-Rodríguez E. (2014). Examining the Relationship between Sex and Motivation in Triathletes. Percept. Mot. Ski..

[35] Malchrowicz-Mośko E., Młodzik M., Banio A., Omorczyk A. (2018). Triathlon jako forma konsumpcji sportowej—Motywacje do udziału. Turyzm.

[36] Roethenbaugh G., Triathlon status in Europe, UK and the World (2014). MultiSport Research Ltd. https://www.multisportresearch.com/blog-1.

[37] Freyer W., Gross S. (2002). Tourismus und Sport-Events.

[38] Summers J.J., Machin V.J., Sargent G.I. (1983). Psychosocial Factors Related to Marathon Running. J. Sport Psychol..

[39] Ridinger L.L., Funk D., Jordan J.S., Kaplanidou K. (2012). (Kiki) Marathons for the Masses: Exploring the Role of Negotiation-Efficacy and Involvement on Running Commitment. J. Leis. Res..

[40] Sharkey B.J. (1997). Fitness and Health.

[41] MacNeill I. (1999). The Beginning Runner’s Handbook.

[42] Malchrowicz-Mośko E., Poczta J. (2019). Motivations for Running in Men: A Comparative Analysis of Local Runners and Sports Tourists. Turyzm.

[43] Poczta J., Almeida N., Rozmiarek M., Młodzik M., Malchrowicz-Mośko E. (2021). Men’s and Women’s Style of Living and Motivation to Run in Charity Events. Sustainability.

